# Reduced Time to Admit Emergency Department Patients to Inpatient Beds Using Outflow Barrier Analysis and Process Improvement

**DOI:** 10.5811/westjem.18626

**Published:** 2024-08-01

**Authors:** Marjorie A. Erdmann, Ipe S. Paramel, Cari Marshall, Karissa LeHew, Abigail Kee, Sarah Soliman, Monica Monica Vuong, Sydney Sydney Spillane, Joshua Joshua Baer, Shania Shania Do, Tiffany Tiffany Jones, Derek Derek McGuire

**Affiliations:** * Oklahoma State University, Spears School of Business, Center for Health Systems Innovation, Stillwater, Oklahoma; † Oklahoma State University Center for Health Sciences, College of Osteopathic Medicine, Tulsa, Oklahoma

## Abstract

**Objective:**

Because admitted emergency department (ED) patients waiting for an inpatient bed contribute to dangerous ED crowding, we conducted a patient flow investigation to discover and solve outflow delays. After solution implementation, we measured whether the time admitted ED patients waited to leave the ED was reduced.

**Methods:**

In June 2022, a team using Lean Healthcare methodologies identified flow delays and underlying barriers in a Midwest, mid-sized hospital. We calculated barriers’ magnitudes of burden by the frequency of involvement in delays. During October–December 2022, solutions targeting barriers were implemented. In October 2023, we tested whether waiting time, defined as daily median time in minutes from admission disposition to departure (ADtoD), declined by conducting independent sample, single-tailed *t*-test comparing pre- to post-intervention time periods, January 1–September 30, 2022 (273 days) to January 1–September 30, 2023 (273 days). Additionally, we regressed ADtoD onto pre-/post period while controlling for ED volume (total daily admissions and ED daily encounters) and hospital occupancy. A run chart analysis of monthly median ADtoD assessed improvement sustainability.

**Results:**

Process mapping revealed that three departments (ED, environmental services [EVS], and transport services) co-produced the outflow of admitted ED patients wherein 18 delays were identified. The EVS-clinical care collaboration failures explained 61% (11/18) of delays. Technology contributed to 78% (14/18) of delays primarily because staff’s technology did not display needed information, a condition we coined “digital blindness.” Comparing pre- and post-intervention days (3,144 patients admitted pre-intervention and 3,256 patients post), the median minutes a patient waited (ADtoD) significantly decreased (96.4 to 87.1 minutes, *P* = 0.04), even while daily ED encounter volume significantly increased (110.7 to 117.3 encounters per day, *P* < 0.001). After controlling in regression for other factors associated with waiting, the intervention reduced ADtoD by 12.7 minutes per patient (standard error 5.10, *P* = 0.01; 95% confidence interval −22.7, −2.7). We estimate that the intervention translated to ED staff avoiding 689 hours of admitted patient boarding over nine months (ADtoD coefficient [−12.7 minutes] multiplied by post-intervention ED admissions [3,256] and divided by 60). Run chart analysis substantiated the intervention’s sustainability over nine months.

**Conclusion:**

After systemwide patient flow investigation, solutions resolving digital blindness and environmental services-clinical care collaboration failures significantly reduced ED admitted patient boarding.

Population Health Research Capsule What do we already know about this issue?

*Boarding admitted patients in the ED threatens patient and staff health. It is at a crisis level in the US, but reducing it has proven difficult.*

What was the research question?

*Can a systemwide patient flow investigation lead to significant reduction in admitted patient boarding time?*

What was the major finding of the study?

*Admitted patient boarding was reduced by 12.7 minutes/patient (P = 0.01; 95% CI −22.7, −2.7). Technology was the primary issue.*

How does this improve population health?

*These findings will help other institutions to reduce ED boarding and preserve community access to emergent care without increasing the cost of care.*

## INTRODUCTION

Emergency physicians in the United States have raised the alarm about dangerous and worsening emergency department (ED) crowding.^1^ ^,^ ^2^ ^–^ ^4^ Crowding is associated with patient harm,^2^ ^,^ ^5^ increased staff stress,^4^ ^,^ ^6^ medical errors,^7^ and patient mortality.^5^ ^,^ ^8^ ^,^ ^9^ Although there is not a single cause for crowding, in large part it occurs when admitted patients are boarded in the ED because access to an appropriate bed is blocked.^1^ ^,^ ^2^ ^–^ ^4^ ^,^ ^10^ ^,^ ^11^ Access block stems from over-capacity units as well as a larger, long-standing issue: inefficient patient flow in US hospitals.^3^ ^,^ ^10^ ^,^ ^12^ ^–^ ^14^ Inefficient patient flow not only bottlenecks patients in the ED but is also known to drive patient outcomes down and costs up,^12^ two problems the US is urgently working to resolve.^15^ Compared to peer high-income countries, the US ranks last in health outcomes^16^ and first in costs.^17^

Considering that reducing ED boarding time is associated with reduced harm to patients and staff and reduced costs,^11^ policymakers responsible for cost and outcome trends and administrators responsible for alleviating crowding are acutely interested in strategies that improve the outflow of admitted ED patients. A major obstacle to improving ED outflow is that few hospitals are adept at patient flow investigations and interventions. Hospitals tend to improve patient flow one department at a time, assuming that these within-department flow efficiencies stack up to overall gains.^13^ Experts warn that this approach can backfire.^13^ ^,^ ^18^ Well-intended department improvement programs can negatively impact patient flow because individuals focus on their own department’s efficiency achievements and do not consider the effect of their actions on upstream or downstream departments.^18^ Moreover, because hospital processes are complex and deeply interlocked,^19^ it is unrealistic for staff in one department to accurately predict or observe unintended outcomes in other areas.

Administrators have tried to decrease boarding times by taking a within-ED improvement approach; however, the interventions have failed and have even worsened boarding.^2^ ^,^ ^11^ For example, Kelen et al’s^2^ literature review found that 1) neither increasing the number of ED staff nor improving ancillary services’ turnaround time had any impact on boarding, and 2) increasing the size of the ED made turnaround time worse. What has worked to improve ED outflow is directly reducing access blocks. One hospital reduced boarding by blocking their surgery department from using a certain number of beds based on a predicted number of ED admissions.^2^ Another hospital took a process-improvement approach and reduced access blocks by simply discharging patients earlier in the day, incrementally improving access by improving processes.^14^

The present study is two-pronged. In Phase 1, we moved away from single-department patient flow investigations and instead conducted a multi-department, systemwide, patient flow study to identify potential process improvements to reduce access delays. In Phase 2, we evaluated the effect of our process improvements on admitted ED patient bed-wait times measured by daily median of admission disposition to departure (ADtoD) minutes comparing pre- and post-process improvement intervention. We hypothesized that a patient flow investigation would reveal delays and that the subsequent process improvement would significantly reduce the time admitted ED patients waited for a bed.

## METHODS

In 2022, a mid-sized, urban-based hospital in the Midwest partnered with a university health innovation center that specializes in workflow design to help resolve admitted ED patient outflow delays, identify solutions, and measure the effectiveness of implemented solutions. The innovation team chose to use Lean Healthcare methodologies. Lean Healthcare has been applied to improve patient care processes and material flows and, to a lesser extent, patient flow.^20^ ^,^ ^21^ Lean is a production improvement process developed by the Toyota Production System (Toyota Motor Corp, Toyota, Aichi, Japan), which has been used in healthcare to increase efficiency, reduce costs, and improve patient outcomes.^22^ Lean provides a practical approach to understanding complex systems.^23^

The innovation center trains first- and second-year medical students in Lean Healthcare, a program designed to teach systems thinking earlier in medical education.^24^ In 2022, a Lean Healthcare expert assembled a team of nine second-year medical students, trained them in Lean, and assigned the current problem to them to investigate and solve collectively. The investigation was deemed non-human subject research by the university institutional review board.

### Observational Field Investigation, Process Mapping, and Delay Identification

The team’s field investigation was conducted in June 2022. Hospital executives introduced the team to employees to ensure frontline worker cooperation. The team collected procedure manuals from departments and created an observation schedule to investigate segments of patient flows from ED admission through discharge. Patient delays in the ED were the longest between 3 pm–8 pm; therefore, that period was prioritized for observation.

The hospital’s technology included the Epic electronic health record system (EHR) and the Epic environmental services system (Epic Systems Corp, Verona, WI) with an Ascom phone integration (Ascom Holding AG, Baar, Switzerland). Over 21 days, the team observed and mapped patient flow, observed workers, and followed patients, noting workarounds, bottlenecks, and coordination points with other departments. Team members discussed processes and patient flow with over 100 staff, including managers, ED staff, physicians, transfer coordinators, environmental services (EVS) staff, transporters, maintenance staff, and receiving floor nurses. The team identified discrepancies between protocols and actual work, gathered time-stamped data, and directly timed some process steps.

The team integrated the processes and mapped the patient journey from ED admission through discharge and the EVS processes used to turn a bed between patient occupancies. The team used “swim lanes,” a technique to map processes occurring simultaneously in different departments. The team created a list of delays; delays were defined as when patient flow stalled for any reason. The swim lanes identified key staff roles across multiple departments that co-produced ED patient outflow.

### Underlying Barrier Categorization and Magnitude of Burden Analysis

Post-investigation, we grouped common issues, named them, and built a conceptual framework representing system-level outflow barriers. We assessed which barriers contributed to each delay and tallied the frequency with which each barrier contributed to delays. We compared each barrier’s magnitude of burden by frequency and percentage (number of delays affected by a barrier/total number of delays); more than one barrier type could be associated with each delay.

### Recommended Solution Prioritization

We designed specific solutions for administrative action, organized them by barrier targeted, and sorted them into a 3 × 2 matrix by the degree to which the solution was deemed controllable (controllable, probably controllable, uncontrollable) and the estimated associated cost (no cost, cost). We relied on the magnitude of the burden to prioritize solutions within the high controllability and low-cost category to prioritize selected solutions. In the fourth quarter of 2022, solutions were implemented.

### Intervention Effectiveness

In October 2023, we used a quasi-experimental design to evaluate whether interventions reduced the time admitted patients waited to leave the ED. Quasi-experimental designs are appropriate to assess interventions without random assignment.^25^ We defined individual-level patient waiting time as the time from when a patient’s disposition became “admitted” in the EHR to when that patient departed the ED, ensuring that we evaluated only admitted patients. From this data, the EHR provided a daily median time of patients’ admission disposition to departure in minutes (ADtoD), and we used the daily median as a per-patient representation to detect waiting differences between two time periods. Medians were both readily available in the EHR and preferred in analysis to avoid artificially high or low daily values caused by outliers.

The two time periods we compared were pre-intervention days (January 1–September 30, 2022) and post-intervention days (January 1–September 30, 2023). We excluded the period from October–December 2022 because it was the period when solutions were being implemented. Matching months (January–September) for both time periods minimizes seasonal effects, which is a concern with ED outcome investigations because ED volumes follow seasonal trends.^26^ The strength of using day-level measures across the two nine-month time periods is that they provide a good sample size (total number of days 546, 273 days in each period) for statistical comparison vs monthly level data (18 months with 9 in each period).

Days were categorized as belonging to either the pre- or post-intervention period samples. Because our hypothesis was that the ADtoD would decrease in the second period, we used a single-tailed *t*-test to compare ADtoD between periods. To describe how the time periods differed in ED volume and hospital capacity (factors that could theoretically affect ADtoD), we pulled data by day for 1) total number of occupied hospital beds, including observation beds; 2) total number of patients admitted from the ED; and 3) total number of ED encounters. In addition to descriptive analytics, we conducted independent sample two-tailed *t*-tests on these three variables to detect period differences. Finally, we used standard least squares regression to test for the effects of the intervention (pre, post) on ADtoD, while controlling for ED volume and hospital occupancy variables.

We used a run chart for a visual, temporal analytic view of improvement to evaluate whether improvement occurred after intervention and to determine whether improvements were sustainable.^27^

## RESULTS

### Observational Field Investigation, Process Mapping, and Barrier Identification

The total personnel cost for the investigation was $16,000. Because the hospital partnered with the university in a joint effort to train medical students in systems-based practice, the investigation came at no cost to the hospital. The university paid each of the nine medical students $12.50/hour for 0.5 full-time equivalent (FTE) for a one-month (80 hours) internship, which totaled $9,000. The Lean consultant was a full-time innovation center employee who we estimated dedicated 0.75 FTE for the month (90 hours) at a cost of $7,000, including salary and benefits.

**Figure 1.**
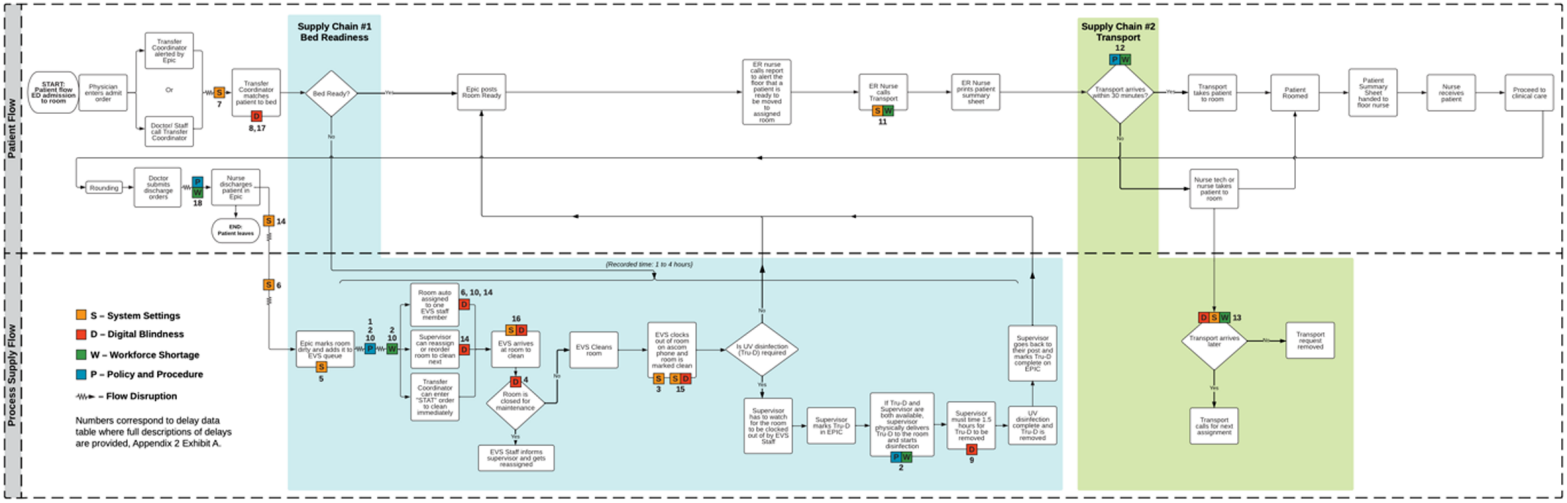
Three-department process map depicting patient- and supply flow.

The team identified that boarded ED patients primarily waited for two supplies from other departments: clean beds provided by EVS; and transport services. The departments’ processes were interlocked; interlocking processes are defined as when one process flow is reliant on/triggered by an output of another process, such as a digital signal or an action. Mapping the supply process for each and their intersection with the ED process generated Figure 1. We called this three-department process map *global bed management*. We categorized flow through bed management into four multi-department collaborative steps: 1) processes used to provide clean, ready beds; 2) processes used to assign patients to open beds; 3) processes used to transport a patient for bed occupancy; and 4) processes used to resupply beds freed from discharge. Across these steps, we logged 18 delays. The location of the delays is depicted in Figure 1. A supplemental file provides a higher quality image; and detailed descriptions of delays are listed in Appendix Exhibit A.

Across the interlocking departments, six staff roles co-produced global bed management whom we named flow facilitators; four were patient care-based (transfer coordinator, transporters, ED nurses, and floor nurses), and two were EVS-based (supervisor and staff). The EVS and clinical care staff depended primarily on the EHR platforms to send digital signals to each other for coordination, in contrast to transport services, which were coordinated by phone. An example of clinical care sending EVS a signal was when floor nurses entered a patient’s discharge into the EHR; that entry triggered the posting of that patient’s bed onto the EVS cleaning queue. In total, 11 of the 18 (61%) delays were caused by EVS-clinical care collaboration failures caused by them not perceiving themselves as a collaborative team. Because they lacked a shared goal to connect patients as efficiently as possible to beds, they lacked communication processes to facilitate that process. Appendix Exhibit A details EVS and clinical care standard work processes that disregarded the effect they had on each other. The EVS staff had no visibility into clinical information due to patient data protection. There was no reason that clinical care staff’s visibility into EVS systems was limited.

### Barrier Types

We identified four common, system-level causes (barriers) for the 18 delays. Two stemmed from technology design (rigid system settings and “digital blindness”) and two stemmed from management issues (workforce shortage and policy and procedures).

#### Technology-based barriers

We found rigid *system settings* embedded in technology applications prevented staff from changing inaccurate information such as when a clean bed was listed as dirty because EVS lacked access to change bed statuses not assigned to them. *Digital blindness,* a term we coined to describe the common condition when staff were unable to see pertinent information due to poor technology design, such as EVS not seeing which beds had waiting patients. In sum, users relied on inaccurate information or made decisions without complete information. Because digital blindness was widespread across clinical care and EVS, we labelled three distinct types: *true status blindness* (eg, being shown the inaccurate status of a bed), *collaboration blindness* (eg, assigning patients to beds without knowing order of readiness, causing patients to exit the ED out of admission order), and *sporadic output blindness* (eg, nurse discharge data entry creating EVS dirty-bed notification in 0–20 minutes).

Delays associated with rigid system settings are designated as “S” in Figure 1 and Appendix Exhibit A. All types of digital blindness are designated as “D” in Figure 1 and Appendix Exhibit A.

#### Management-based barriers

Transporters, clinical care, and EVS staff suffered from understaffing due to *workforce shortages,* a management issue constrained by local resources.^28^ ^–^ ^30^ *Policy and procedures* contributed to delays in two ways. First, in all cases except one the delays associated with workforce shortage were also associated with policy and procedure issues (Appendix Exhibit A). We concluded that policy and procedures can reflect an assumption of an optimal workforce supply, and adherence to them during shortages can backfire and exacerbate delays. Consider one ED policy that required ED nurses to wait 30 minutes after calling transport services before using their own techs for transport. Adherence created 30-minute delays when transporters were short-staffed and ED techs were available to transport.

Second, we discovered within-department procedures that caused delays in other areas. For example, prior to intervention, EVS procedures included all staff changing shift at the same time and conducting a brief meeting at shift change. To accommodate both shift change and the meeting, staff stopped cleaning the queued beds for one hour every day to wind down their cleaning work pre-shift change, to attend the meeting, and to ramp it up post-shift change. Compounding this problematic procedure, it occurred at 3 pm, helping to explain why ED patient waiting increased at 3 pm each day. The EVS procedures weren’t the only problematic ones; others included allowing nurses to delay discharge entry, thus eliminating timely signals to EVS of dirty beds and no transporter cancellation policy causing transporters to waste time looking for already transported patients.

Delays associated with workforce shortage are designated with a “W” in Figure 1 and Appendix Exhibit A. Delays caused by policy and procedures are designated with a “P” in Figure 1 and Appendix Exhibit A.

**Figure 2.**
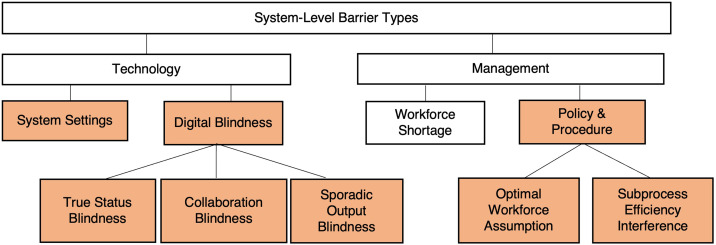
System-level barrier types. Barriers unknown before the investigation are shaded.

Figure 2 summarizes the system-level barriers and underlying causes. Of these, only workforce shortage was a recognized cause for delays before our investigation.

#### Magnitudes of burden from barrier types

**Figure 3.**
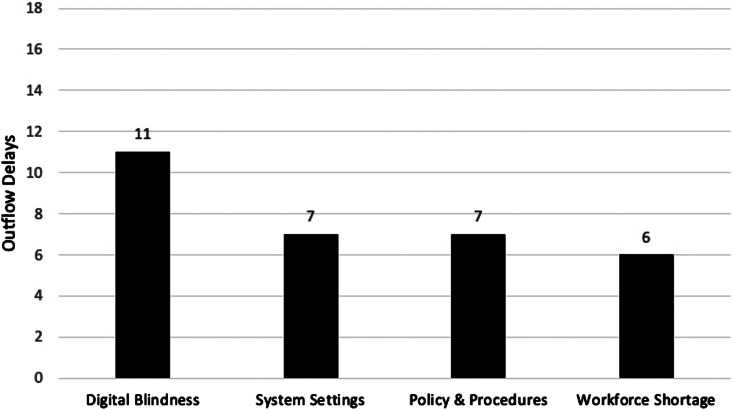
Comparison of barrier types’ magnitude of the burden on emergency department patient outflow by frequency of barrier contribution to outflow delays.

Using the delay data compiled and available in Appendix Exhibit A, we found that technology-based barriers (barriers associated with digital blindness or system settings) were twice as prevalent (14/18; 78%) compared to management-based barriers (barriers associated with workforce shortage or policy and procedures) (7/18, 39%). Digital blindness was associated with the most delays at 61% (11/18), followed by system settings 39% (7/18), policy and procedures 39% (7/18), and workforce shortage 33% (6/18) (see Figure 3).

### Solution Generation and Selection

The Lean team generated and evaluated 25 solutions (Appendix Exhibit B). Over half of the identified solutions (59%; 13/22) were considered controllable and estimated to have no cost based on information technology (IT) capabilities. Solutions with costs reflected the need to hire staff, which may or may not have been viable due to local shortages.^28^ ^–^ ^30^

For solution selection, we turned to the magnitude of burden calculation pointing to digital blindness, system settings, and policy and procedure issues, each of which had a no-cost solution. The following solutions were implemented in the fourth quarter of 2022: 1) the IT department corrected the system setting that prevented EVS staff from updating dirty rooms to be cleaned by allowing EHR access to the electronic processes that assigned EVS staff to rooms—access that resolved transfer coordinator blindness to true bed status; 2) IT further decreased transfer coordinators’ digital blindness by creating visibility in the EVS cleaning queue, thereby revealing the likely order of available beds; and 3) EVS management changed departmental policy and procedures by staggering EVS staff shifts, to ensure continuous progress in ready bed supply, and eliminating all-staff daily meetings. The effects of individual solutions were not measured. Collectively, these solutions were the intervention.

### Intervention Effectiveness

We completed all statistical analyses with JMP Pro 16 software (SAS Institute, Cary, NC). Sample 1 (year 2022 pre-intervention) and Sample 2 (2023 post-intervention) each had the same sample size (273). Descriptive statistics and independent sample *t*-test results are presented in Table 1.

**Table 1.** *T*-test results of pre- and post-intervention.

	Sample 1: pre-intervention days Jan–Sep 2022 (*n* = 273)		Sample 2: post-intervention days Jan–Sep 2023 (*n* = 273)							
	M	SD	M	SD	Diff	df	t	SE	95% CI	Single-tailed *P*
ADtoD	96.4	55.6	87.1	63.3	−9.2	535.99	1.80	5.1	−19.3 – 0.8	0.04
										Two-tailed *P*
Daily total admitted ED patients	11.52	4.04	11.93	4.08	0.41	543.95	1.18	0.34	−0.27 – 1.93	0.24
Daily total ED encounters	110.72	18.39	117.28	14.23	6.56	511.82	4.66	1.40	3.80 – 9.33	<0.001
Bed occupancy	53.58	11.15	54.19	10.61	0.61	542.66	0.66	0.93	−1.22 – 2.44	0.51

*ADtoD*, admission disposition to departure; *Diff*, mean difference; *df*, degrees freedom; *SE*, standard error; *CI*, confidence interval; *ED*, emergency department.

ADtoD (the median number of minutes a patient waited each day) was significantly lower in the post-intervention period (*P* = 0.04), while the ED staff managed substantially higher average daily ED encounters (*P* < 0.001). The number of daily admits did not significantly differ (*P* = 0.24), nor did the daily census (*P* = 0.51). Notably, after the intervention, a busier ED achieved lower ADtoD times. Regression of ADtoD median time onto pre/post categorization while controlling for three other independent variables reveals that the intervention significantly reduced ADtoD times by 12.7 minutes (*P* = 0.01). Table 2 shows the intervention’s effect of admission disposition to departure median times (ADtoD) while controlling for bed occupancy by day, total admitted ED patients by day, and total ED encounter by day.

**Table 2.** Standard least squares regression model results (*N* = 576).

ADtoD predictors	Estimate	SE	*P*	95% CI
Bed occupancy	−0.33	0.24	0.17	−0.81 – 0.14
Total admitted ED patients	2.38	0.69	0.001	1.02 – 3.73
Total ED encounters	0.41	0.17	0.01	0.09 – 0.74
Pre-post	−12.7	5.1	0.01	−22.7 – −2.7

*ADtoD*, admission disposition to departure; *SE*, standard error; *CI*, confidence interval.

To estimate the intervention’s effect on ED staff’s exposure to admitted patient boarding, we multiplied the ADtoD coefficient (−12.7 minutes) representing each patient’s reduced waiting time by the number of post-intervention ED admissions (3,256) and divided by 60 to convert to hours, resulting in an estimation that ED staff avoided 689 hours of ED boarding over the nine-month post-intervention period.

**Figure 4.**
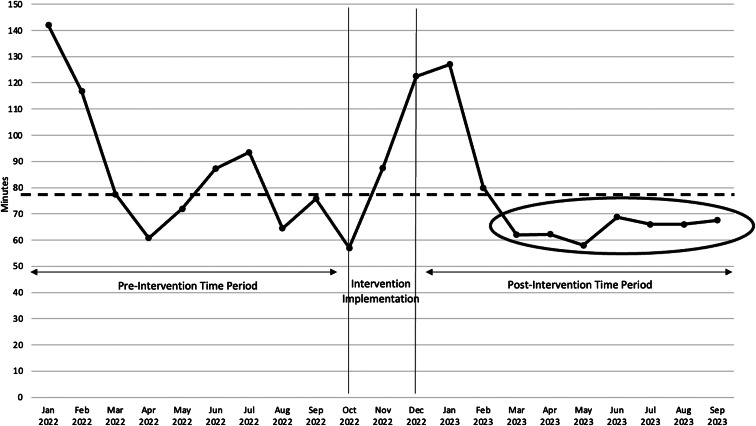
A run chart depicting the admitted patient ED outflow process improvement with sustained median time under pre-intervention baseline.

In addition to statistical significance analyses supporting the effectiveness of the intervention, Figure 4 depicts a run chart supporting that improvement occurred after the intervention and the intervention achieved a shift, a run of at least six sub-baseline values.^27^ Baseline is the pre-intervention period median ADtoD. Because run data must be interpreted in context,^27^ we interpreted January ADtoD increases as reflecting peak hospital occupancy, the effect of which was reduced in January 2023 by the intervention but not eliminated. By February 2023 the median ADtoD approached baseline; afterward, improvement was sustained.

## DISCUSSION

The findings from this investigation indicate that emergency physicians and their patients were enduring unnecessary outflow delays discoverable through a systemwide patient-flow investigation. Although over-capacity inpatient units tend to dominate discussions about ED boarding,^2^ ^–^ ^4^ ^,^ ^11^ we discovered delays buried in technology and within-department procedures. In Phase 1, the use of Lean methodologies revealed the outflow process and its dependence on global bed management (Figure 1), flow facilitators and their reliance on technology to collaborate, and the system-level root causes driving delays (Figure 2). The investigation led to designing low-cost solutions that in Phase 2 achieved administration’s goal of reducing admitted patient boarding in the ED.

The intervention consisted of three solutions geared to improve EVS-clinical care collaboration. *T*-test analysis of pre- vs post- intervention periods showed that ADtoD was significantly decreased while the ED was significantly busier (Table 1). Regression analysis controlling for effects from hospital capacity and ED volume demonstrated that the intervention decreased the median ADtoD by nearly 13 minutes (Table 2). Because we estimate that the ED staff avoided 689 hours of ED boarding in the post-period, we contend that the intervention substantially benefited staff. Finally, charting the monthly median ADtoD trend substantiated that improvement occurred after the intervention period and was sustainable (Figure 4).

Not surprisingly, given the widespread healthcare staffing shortages of 2022,^28^ ^–^ ^30^ *workforce shortages* affected outflow. Patients waited for understaffed transporters to move them, understaffed nurses to complete discharges, and understaffed EVS to clean rooms. However, our analysis showed that those shortages had the lowest magnitude of burden on outflow compared to readily solvable issues: *digital blindness; system settings; and policy and procedures* (Figure 3). Before the present investigation, no one at the hospital had ever seen the inter-related, multi-department workflows necessary to move an admitted patient from the ED to a clean bed. The flow map and analysis shifted administration strategies for alleviating boarding from saddling frontline workers to solve to making these workflow adjustments management decisions.

The degree to which technology *hindered* patient flow was unexpected. Although research shows that nurses have called for new, effective technology tools to manage the patient flow for years^31^ and some initiatives have been created,^18^ ^,^ ^32^ ^,^ ^33^ our research uncovered shortcomings of existing tool design, an issue that had gone unnoticed. The upside to discovering problems with existing technology was the availability of low- and no-cost solutions.

Although EHRs are the complex backbone of hospital processes, their embedded processes were unquestioned by staff and untested for optimization by administration. Although multiple systematic reviews have reported on causes of ED crowding,^2^ ^,^ ^3^ ^,^ ^11^ none suggested that the efficiency of technology processes be tested. The reality of how much staff relied on technology to collaborate with others made *digital blindness* the largest barrier. Digital blindness is an issue that has gone unnamed; thus, the magnitude of its effects on other processes, staff, and patients has gone unmeasured. Defining *digital blindness* opens the issue for practical exploration, future research, and innovation design.

The present study results also spotlight the importance of EVS, which has been overlooked in current research. Although EVS is mentioned in a few patient-flow improvement studies,^18^ ^,^ ^32^ in no case was EVS-clinical care coordination central or emphasized. Policymakers should take notice of how, in this study, within-hospital integration lapses were eroding care and productivity. Although the integration of health information systems between hospitals and clinicians monopolizes initiatives across federal agencies (ie, Agency for Healthcare Research and Quality,^34^ Centers for Disease Control and Prevention,^35^ Centers for Medicare & Medicaid Services^36^), we call attention to how within-hospital integration issues are threatening patient outcomes and stressing staff.

Insights into ED outflow barriers are timely for practical application. There are discussions that the ED boarding time standard will be lowered from four to two hours, a 50% reduction.^2^ When administrators seek strategies to meet this aggressive reduction in boarding, we recommend analyzing the bed management cycle and processes with Lean methodologies. Our process uncovered delays unnoticed by any single department, avoided individual patient variabilities that can derail flow investigations,^8^ found problematic within-department efficiency solutions, and was appreciated by frontline staff. Administration reported to the innovation team that they believed the investigation improved morale and created enthusiasm for the subsequent solution implementations because the results explained confusing workplace experiences: why staff did not know what was taking so long for ED patients to flow to floor beds; why EVS staff were not cleaning a dirty room; why a clean room would go vacant; and why similar ED patients exited out of order of admission.

## LIMITATIONS

The main strengths of this study are its complete review of processes and departments affecting ED patient outflow and the durability of the intervention’s gains. However, the generalizability of our findings is limited because it is a single-site study of a mid-sized hospital operating one EHR system during a one-month timeframe. Data was not tested with time series modeling, which could provide additional temporal insights not provided by pre/post design. A limitation to replicability is a hospital’s access to Lean Healthcare training, which could drive up the price of the intervention but may be justified given how quickly the team could practically apply new Lean skills. Moreover, the solutions identified do not address other issues driving crowding, such as high community demand for ED services.

## CONCLUSION

A systemwide patient-flow investigation at a single hospital used Lean methods, which proved effective in identifying the barriers that increased the time admitted ED patients waited for access to beds. The barriers were system-level issues (technology, workforce shortage, and policy and procedures); the greatest was technology. Given healthcare systems’ dependence on technology and the crisis level of ED boarding, this study calls for multicenter regional and national research to understand to what extent within-hospital technology integration lapses are blinding staff and eroding care. Meanwhile, administrators should test how their technology supports (or hinders) environmental services-clinical care coordination and be wary of the effect of *within-department* efficiency gains on patient flow. Because the cost of the investigation was low and we were able to generate solutions for flow barriers with little to no associated costs, we conclude that these types of investigations can reduce ED crowding by moving admitted patients out of the ED more quickly without escalating the cost of care.

## Supplementary Information



