# Supplementary material for: Reduced Time to Admit Emergency Department Patients to Inpatient Beds Using Outflow Barrier Analysis and Process Improvement
Source: West J Emerg Med. 2024 Aug 1;25(5):748–57. doi: 10.5811/westjem.18626 (PMC11418872; doi:10.5811/westjem.18626)
Supplement: Supplementary file 1 [file wjem-25-748-s001.docx]

**APPENDIX**

**Exhibit A.** Delay sources are organized by steps in the global bed management process, including the identification of flow facilitators affected by each delay and the underlying barrier type of each delay.

|  | Main findings of delay sources by global bed management process | | EVS-clinical collaboration breakdown | Barrier type | Hrs |
| --- | --- | --- | --- | --- | --- |
|  | | Step 1: Bed readiness | | | |
| 1 | The daily EVS staff shift change huddle halted bed cleaning; the impact was compounded by shift change occurring at peak discharge time. | | P | P | 1 |
| 2 | EVS staff turnover was highest for afternoon shift/peak demand times; there was no staffing prioritization for peak times. | | P | P, W | - |
| 3 | Two-person EVS teams (trainer and trainee) could not indicate that a 2-person team cleaned a room; their fast times would drive down overall cleaning times, creating concerns about the quality of cleaning; so, as a workaround, they were allowed to post them as clean after the appropriate time had passed. | | P | S | 0.5 |
| 4 | Maintenance system was disconnected from the EVS system, so beds waiting for maintenance appeared on the EVS queue without a maintenance hold indication. | |  | D | - |
| 5 | Automated EVS system created bed-cleaning queue without regard to patients waiting for a bed to be cleaned, the time of their wait, or special needs. | | P | S | - |
| 6 | EVS system lacked the ability for overnight room cleaning to communicate the cleaning standard met (discharge or daily clean). | |  | D, S | - |
|  | | Step 2: Patient-bed matching/assigning | | | |
| 7 | EHR presented beds available for patient assignment from discharge entry; so dirty beds were made available for patient assignment. | | P | S | 4 |
| 8 | Transfer coordinator could not see the EVS queue; so they could not predict the order of dirty rooms becoming clean. | | P | D | - |
| 9 | Some rooms required a UV disinfection process. EVS ran equipment but lacked notification after disinfection was completed. | |  | D | 3 |
| 10 | Only EVS supervisors could see bed assignments and expedite or move up rooms in the queue. Due to staff shortages, supervisors worked on the floor, unable to view and edit the queue. | | P | D, P, W | - |
|  | | Step 3: Patient transport | | | |
| 11 | Transport staff shortages were not visible to nurses, limiting the ability to reassign transporters to available ED techs proactively. | |  | D, W | 0.5 |
| 12 | Transport coordination policy instructs nurses to wait 30 minutes after transport request. Nurses started solving transport delays after 30 minutes. | |  | P, W | 0.5 |
| 13 | Patients transported via ED staff after 30 minutes, at times, result in transport arriving to already transported patients. | |  | D, P, W | - |
| Step 4: Bed release from discharge | | | | | |
| 14 | Intermittent delays in clinical staff posting a discharge caused EVS to not know about a room that needed cleaning. | | P | S, D | 0.3 |
| 15 | Proactive EVS staff who clean a room they find in need or were asked to clean were unable to digitally communicate it as clean because the room had not been assigned to them. | | P | S, D, P | 1 to 8 |
| 16 | EVS staff waste time traveling to rooms already cleaned. | |  | S, D | - |
| 17 | Unassigned cleaned rooms waiting on the EVS process (for clean rooms to be assigned before posting clean) are not visible to the transfer coordinator. | | P | D | - |
| 18 | Nurses occasionally delay entering a discharge (eg, to delay admission during clinical handoffs to batch work), which unnecessarily delays EVS notification of rooms to be cleaned. | | P | P, W | - |

*EHR*, electronic health record; *EVS*, environmental services.

Abbreviations for barrier type: *D*, digital blindness; *P*, policy and procedures; *S*, system settings; *W*, workforce shortage.

Delay sources are numbered 1-18, and those numbers are marked on the Appendix Exhibit A process map to show exactly where in flow each delay existed.

*Source*: Authors’ analysis of 2022 investigation of patient flow and flow barriers at a mid-sized Midwestern hospital.

**Exhibit B.** Matrix of delay solutions, categorized by barrier type and sorted to level controllable and cost.

|  | Controllable | Probably controllable | Uncontrollable |
| --- | --- | --- | --- |
| No Cost | *System settings*   - Change EVS settings to allow staff to self-assign dirty rooms. - Change EVS settings to allow 2-person teams to post fast times without creating an alert. - Create a disinfection timer for EVS.   *Digital blindness*   - Give the transfer coordinator access (visibility and/or ordering) to the EVS queue. - Allow transfer coordinator to prioritize rooms for maintenance. - Ensure program updates for platforms are completed.   *Workforce shortage*   - Eliminate ED calls to Transport; rely on ED techs to move patients. - Shift a morning staff to afternoons until EVS hires and onboards.   *Policy and procedure*   - Change the EVS huddle time to avoid delays during peak discharge times. - Stagger EVS shift start times so there is no downtime. - Change the ED protocol to reflect Techs to transport from ED. - Discourage batch discharge of patients; educate why. - Train nurses on EVS protocols and coordination points. | *System settings*   - Change the EVS cleaning queue algorithm in Epic to order rooms by patients’ waiting time (not by dirty-bed notification time). - Eliminate sporadic output.   *Workforce shortage*   - Shift EVS to clean occupied rooms in the early evening when possible instead of in the morning.   *Policy and procedure*   - Create EVS – Transfer coordinator 2-way messaging system; train on protocols. |  |
| Cost | *Workforce shortage*   - Hire or schedule designated discharge nurses. - Hire more EVS staff. - Hire more case managers. | *Digital blindness*   - Integrate the maintenance software to notify the system/flow facilitators of rooms offline due to repairs. | *Workforce shortage*   - Eliminate travel nurses and, thereby, the variables they introduce. |

*ED*, emergency department; *EVS*, environmental services.

*Source*: Authors’ analysis of 2022 investigation of patient flow and flow barriers at a mid-sized Midwestern hospi
